# Supplementary material for: Impact of active case finding for tuberculosis with mass chest X-ray screening in Glasgow, Scotland, 1950–1963: An epidemiological analysis of historical data
Source: PLoS Med. 2024 Nov 5;21(11):e1004448. doi: 10.1371/journal.pmed.1004448 (PMC11537369; doi:10.1371/journal.pmed.1004448)
Supplement: S1 Text — (DOCX) [file pmed.1004448.s003.docx]

**S3 Text: Additional methods**

Annual tuberculosis case notification rates are estimated using the following model:

$$C_{i} \sim NegBin(m_{i},\phi)$$

$$log(m_{i} )=\log(N_{i}) + (\beta_{ACF(i)}+Z_{w(i),ACF(i)}) + (\alpha_{ACF(i)} + U_{w(i),ACF(i)})y_{i}$$

$$V = \left[ \begin{matrix} Z & U \end{matrix} \right]\in\mathbb{R}^{K\times6}$$

$$V \sim MVN(\mu,\Sigma)$$

$$\mu\sim N(0,1)$$

$$\Sigma\sim diag(\tau) \Omega diag(\tau)$$

$$\Omega\sim LKJ(4)$$

$$\beta_{j} \sim N\left( 0,1 \right)$$

$$\alpha_{j} \sim N(0,1)$$

$$\tau_{k} \sim Exp(1)$$

$$\phi\sim Gamma\left( 0.01, 0.01 \right)$$

Where i ranges over 1,…,N indexing each row/datapoint; w() maps each row onto its ward (of which there are K); ACF() maps each row onto its time period: one of ‘pre’, ‘during’, ‘post’. The first line represents the data likelihood, with the data modelled as following a negative binomial distribution, with a single dispersion parameter $\phi$ modelled with a gamma prior. The second line models the mean of the negative binomial distribution in terms of global effects representing the level and trends during the 3 ACF periods ($\alpha$ and $\beta$ respectively), as well as ward-level random effects in the levels (the matrix $Z$) and trend (the matrix $U$). The population offset term, $log(N_{i})$, ensures we are modelling per capita rates. The intercept ($\beta$) and slope ($\alpha$) coefficients are modelled as having normal priors. The remaining equations jointly model the random effects for the levels and slopes in each ward during each of the 3 ACF period as a 6-dimensional multivariate normal (MVN). This approach allows for correlations between the level and slope random effects across ACF periods. The mean for top level MVN is modelled as having an iid normal prior, and the MVN covariance matrix is modelled using the Lewandowski-Korowicka-Joe (LKJ) distribution recommended for modelling covariances in Stan (the distributions specified for $\Sigma$, and the additional random variables $\Omega$ and $\tau_{k}$).

*Estimates of incidence and case detection*


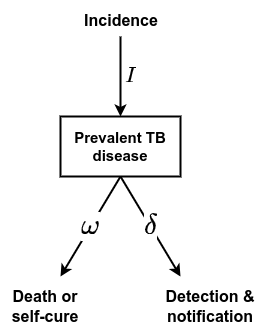


We used a simple competing hazard model of TB detection. Incident TB disease is the inflow to the prevalent pool; competing hazards of detection/notification ($\delta$) and deaths/self-cure ($\omega$) apply to the prevalent pool. The inverse of the rate of death/self-cure represents the mean duration of TB in the absence of treatment:

$$T = \omega^{-1}\approx3 y,$$

the estimate of a 3 years duration based on Tiemersma et al.

Under an equilibrium assumption, we can write down formulae for the case detection ratio (CDR), and in terms of prevalence ($P$) and notifications ($N$):

$$\delta=\frac{N}{P}$$

$CDR=\frac{\delta}{\delta+\omega} \Rightarrow odds(CDR)=T\times\delta\Rightarrow odds(CDR) = T\times N/P$ [✱]

Under the assumption that the increase in notifications ($N_{acf}-N_{pre}$) from just before ($N_{pre}$) to during ACF ($N_{acf}$) represents a coverage fraction $cov$ of the prevalent pool being found:

$$\left( N_{acf}-N_{pre} \right)=P\times cov$$

We can use this together with equation for $CDR$ above to find

$$\boldsymbol{odds(CDR) =}\frac{\boldsymbol{T\times cov}}{\left( \boldsymbol{N}_{\boldsymbol{acf}}\boldsymbol{/}\boldsymbol{N}_{\boldsymbol{pre}}\boldsymbol{-1} \right)}$$

The incidence prior to ACF ($I_{pre}$) can then be estimated as:

$$I_{pre} = \frac{N_{pre}}{CDR}$$
