# Supplementary figures and images for: Impact of active case finding for tuberculosis with mass chest X-ray screening in Glasgow, Scotland, 1950–1963: An epidemiological analysis of historical data
Source: PLoS Med. 2024 Nov 5;21(11):e1004448. doi: 10.1371/journal.pmed.1004448 (PMC11537369; doi:10.1371/journal.pmed.1004448)

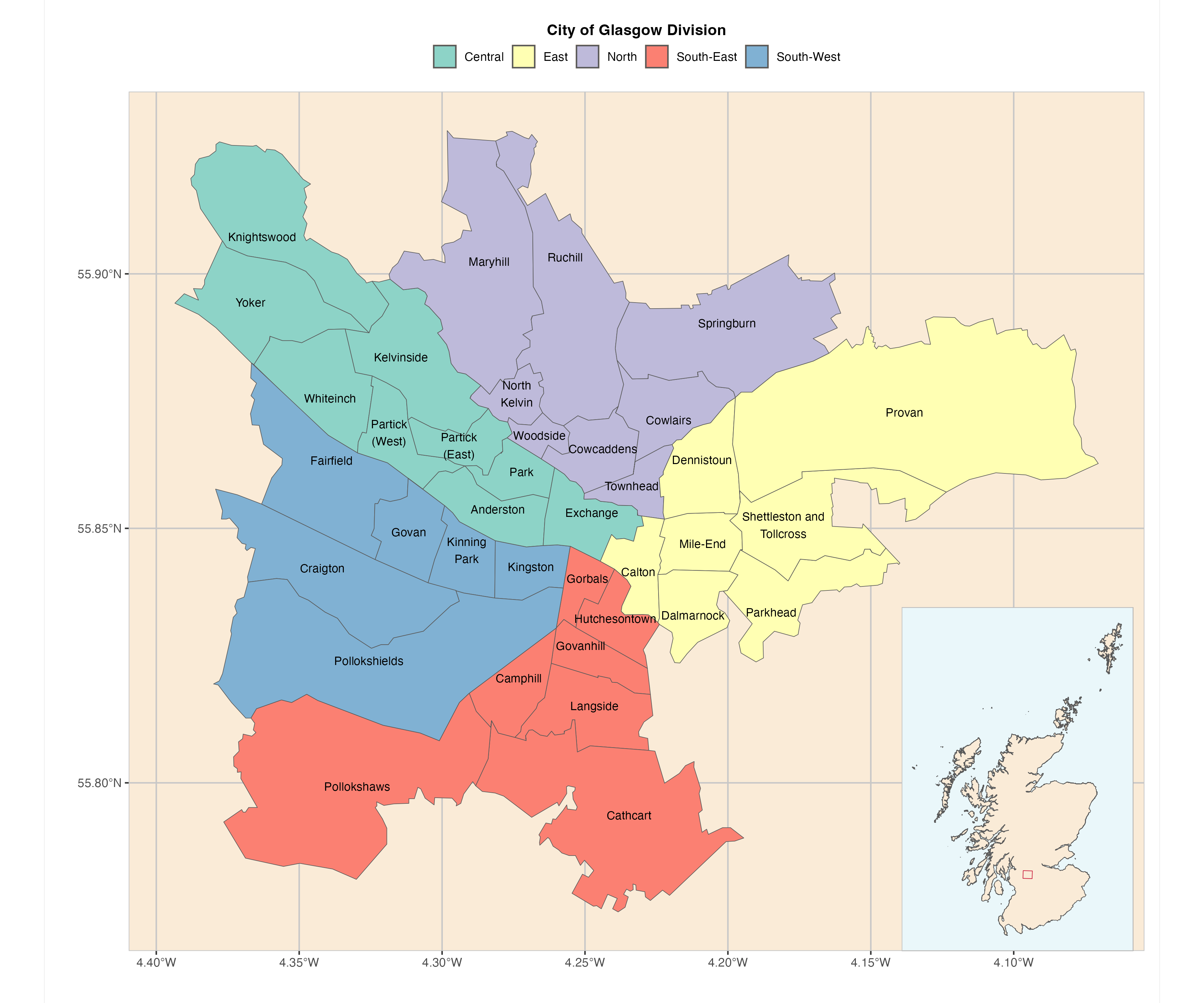

Supplement: S1 Fig — Red box in inset map of Scotland shows location of the main figure (City of Glasgow). Map of Scotland from the UK Office for National Statistics Open Geography Portal (https://geoportal.statistics.gov.uk/datasets/ons::countries-december-2023-boundaries-uk-bfc-2/about), licensed under the Open Government Licence v.3.0 (https://www.nationalarchives.gov.uk/doc/open-government-licence/version/3/). City of Glasgow ward boundaries obtained from a scale 1951–1952 Post Office Directory map drawn by John Bartholomew FRSG held within the City of Glasgow Archive Special Collections (item PSI-52), digitalised using QGIS 3.34.1. (TIFF) [file pmed.1004448.s004.tiff]

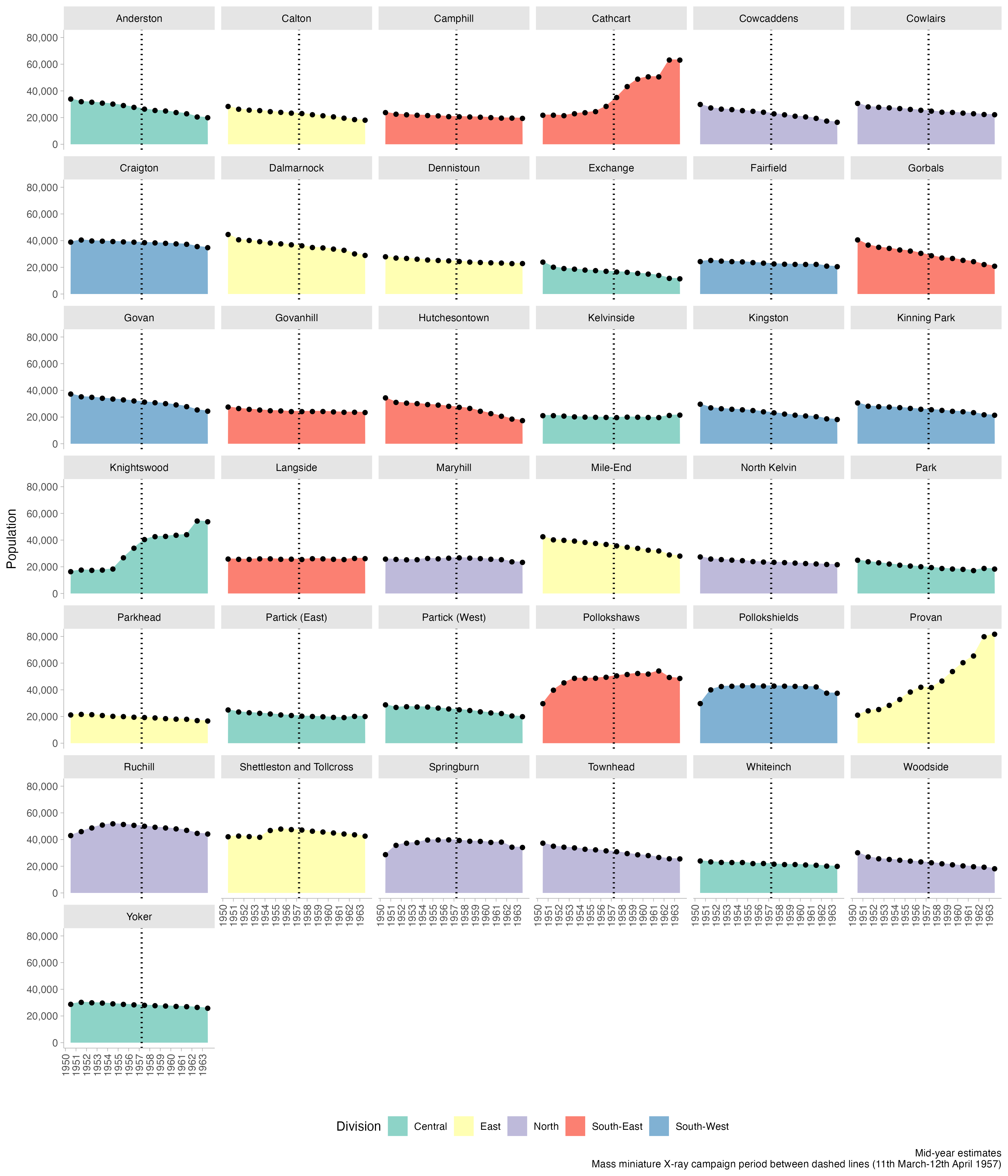

Supplement: S2 Fig — (TIFF) [file pmed.1004448.s005.tiff]

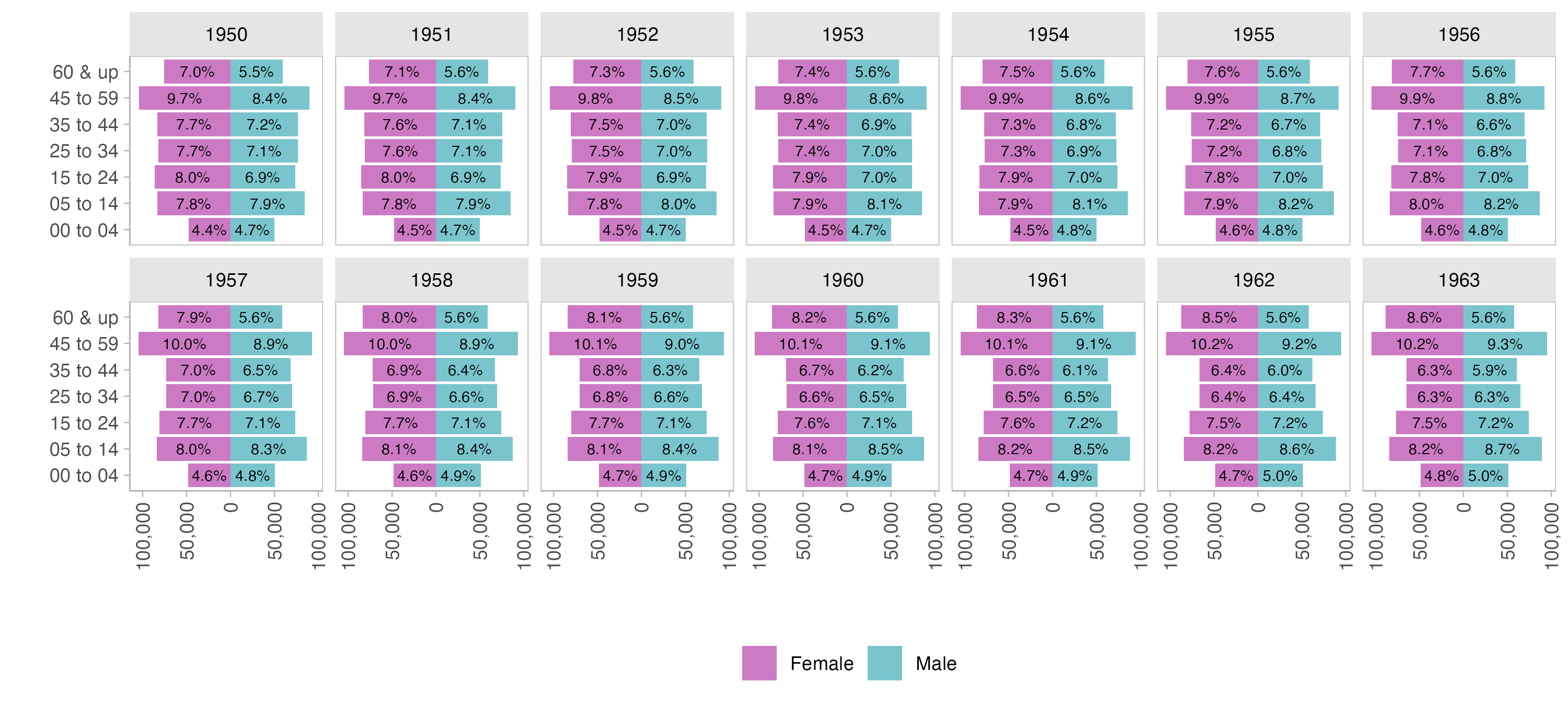

Supplement: S3 Fig — (TIFF) [file pmed.1004448.s006.tiff]

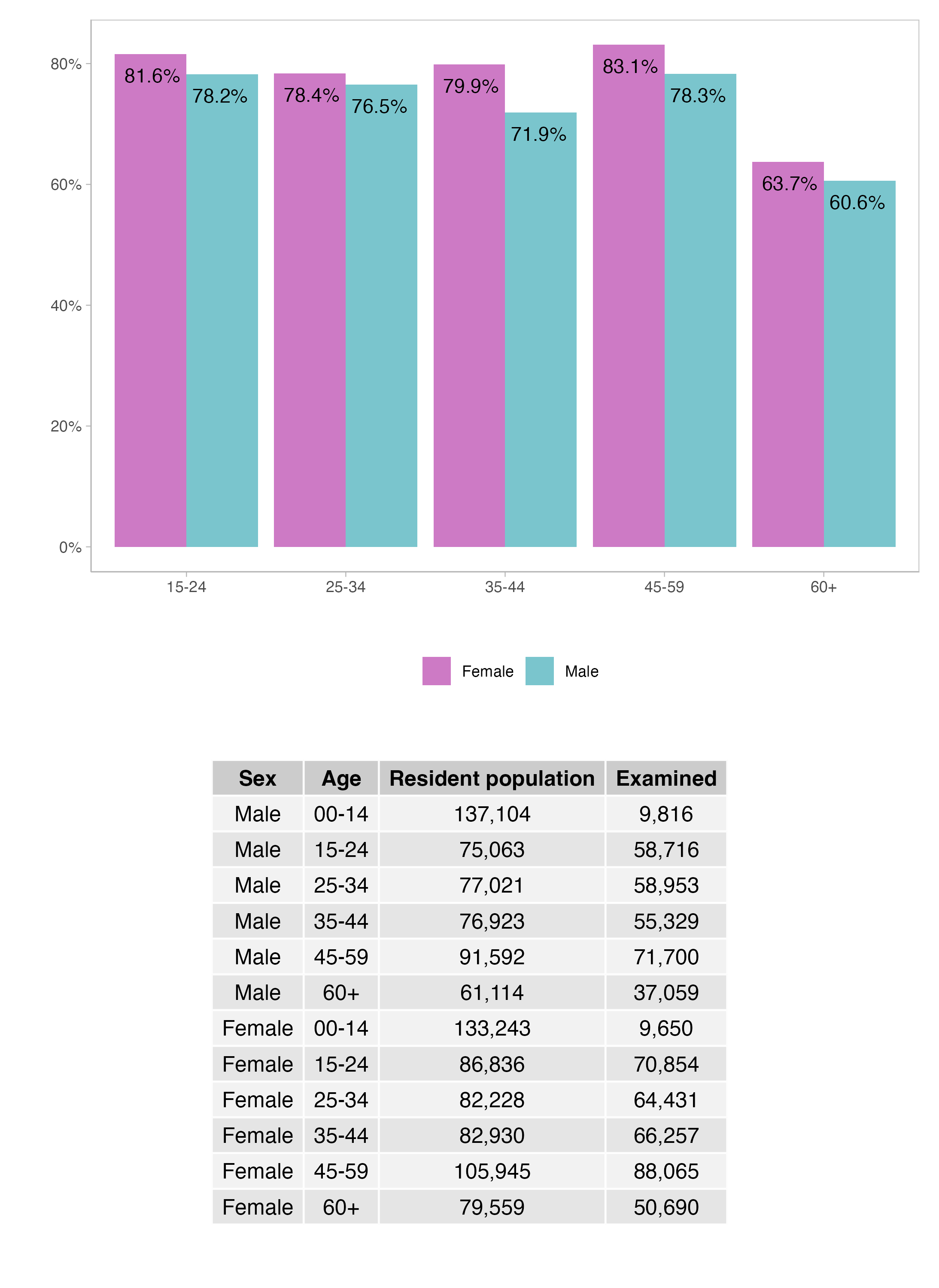

Supplement: S4 Fig — (TIFF) [file pmed.1004448.s007.tiff]

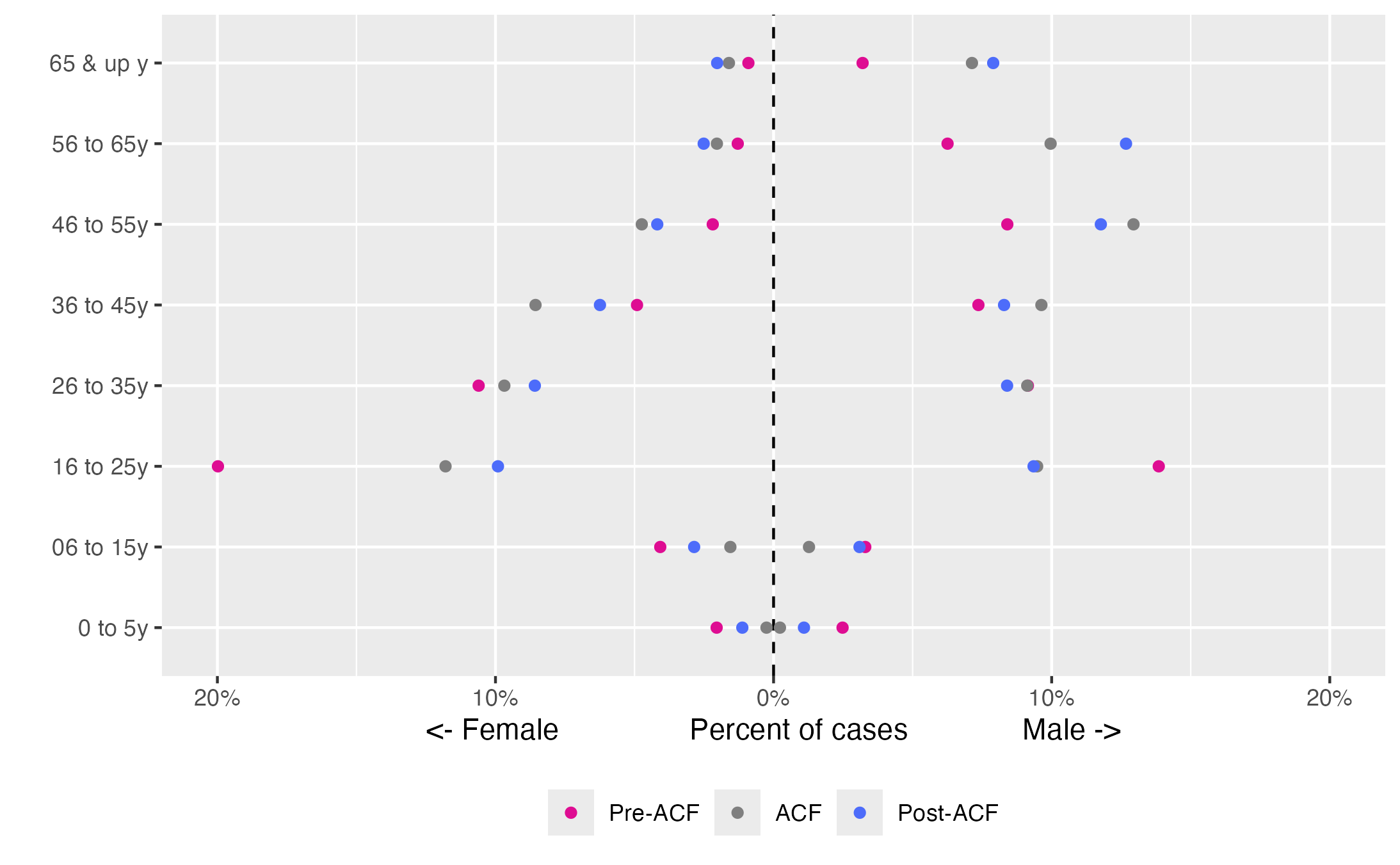

Supplement: S5 Fig — ACF: active case finding. (TIFF) [file pmed.1004448.s008.tiff]

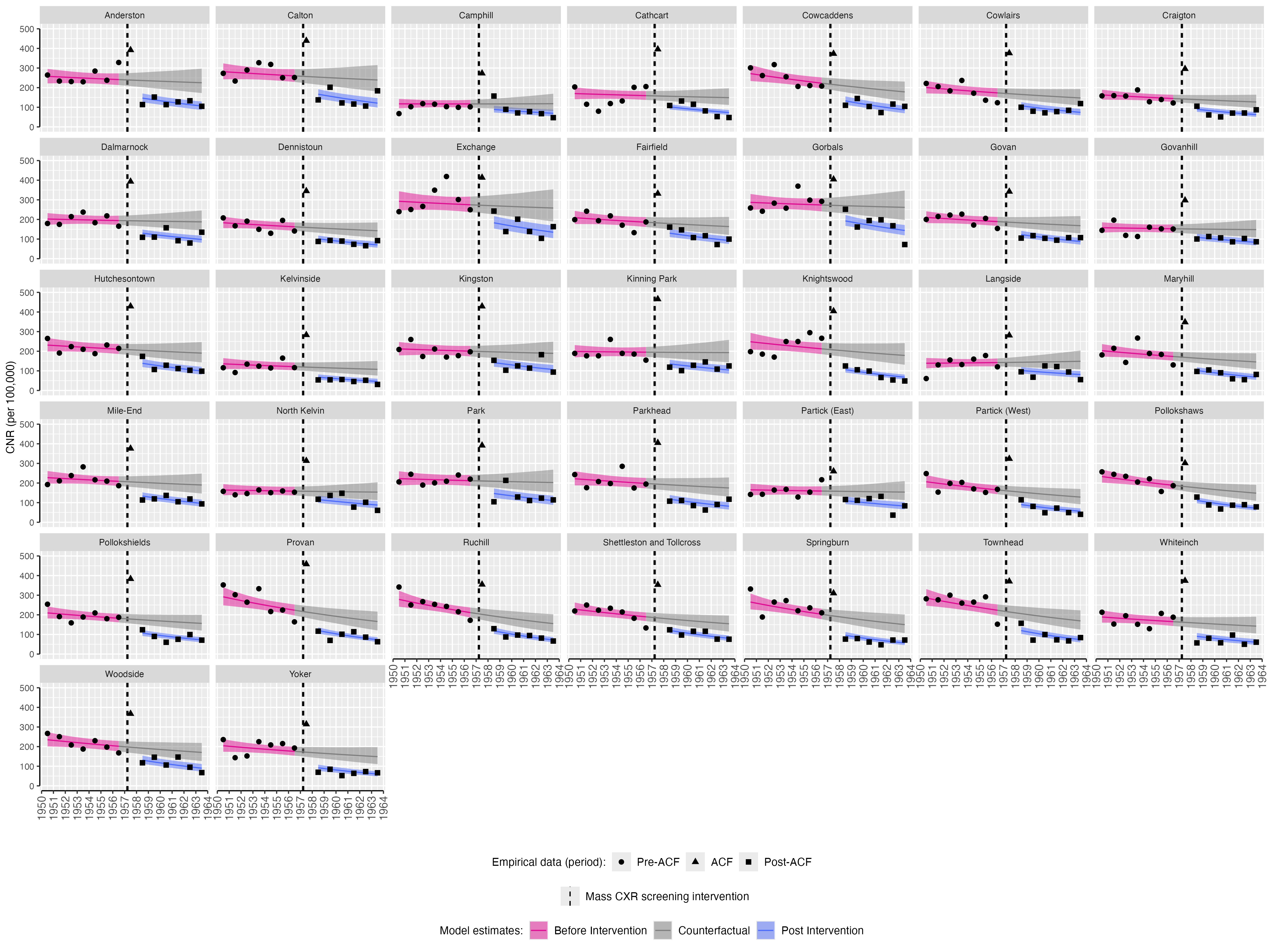

Supplement: S6 Fig — Empirical and modelled case notification rates (per 100,000 population) by ward, with counterfactual of no active case finding intervention. The mass miniature X-ray active case finding campaign occurred between dashed lines (11th March–12th April 1957). CNR: case notification rate. ACF: active case finding. (TIFF) [file pmed.1004448.s009.tiff]

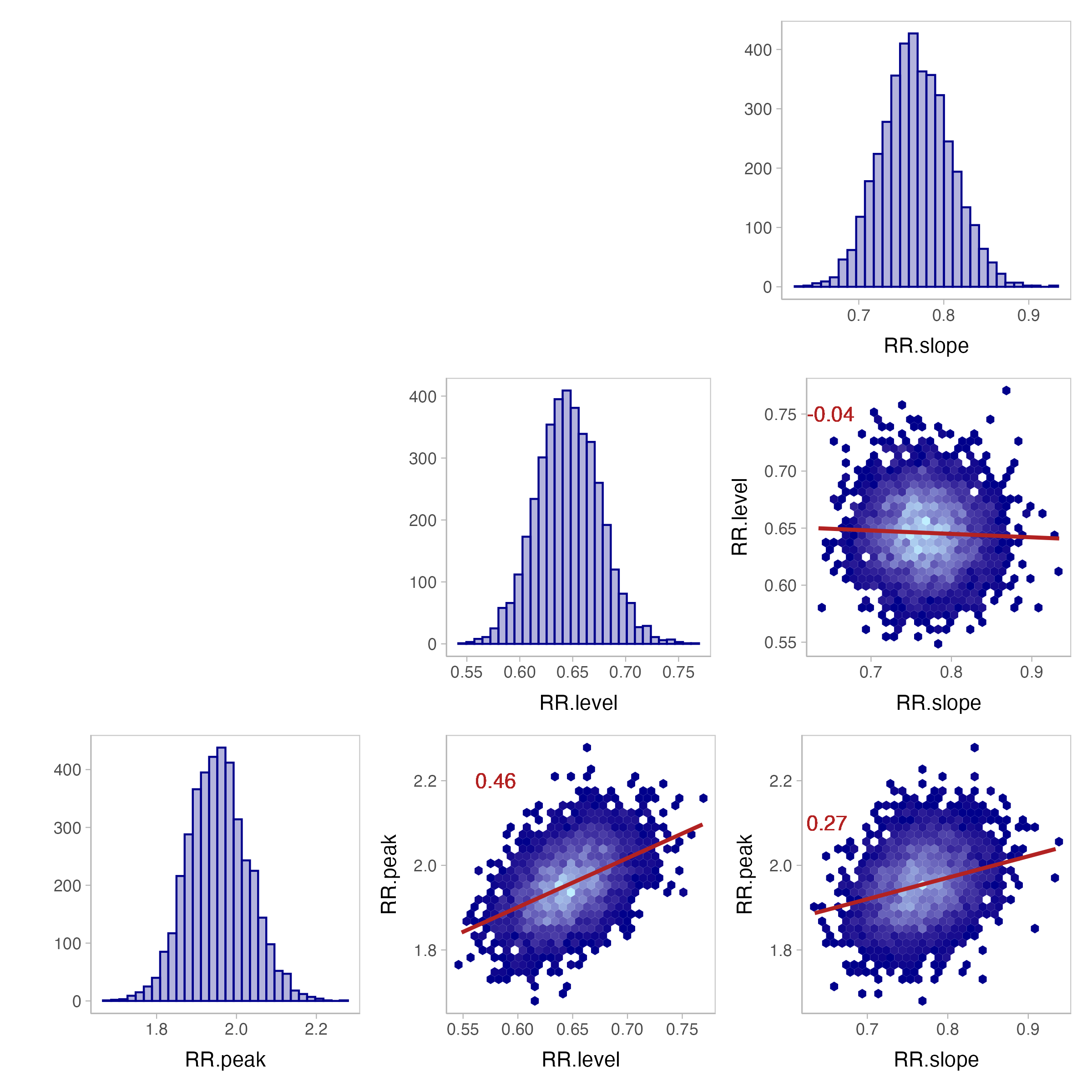

Supplement: S8 Fig — (TIFF) [file pmed.1004448.s011.tiff]

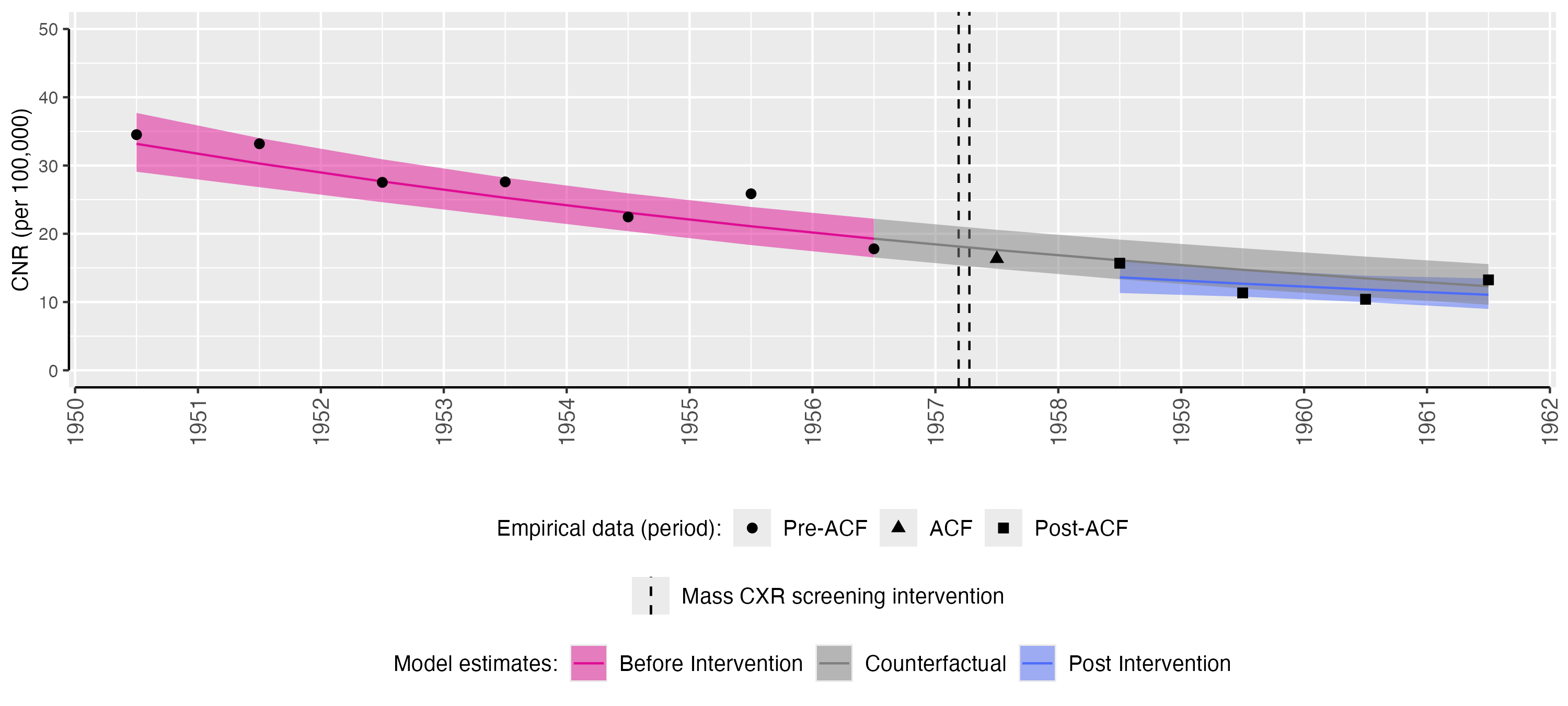

Supplement: S9 Fig — Empirical and modelled case notification rates (per 100,000 population) by ward, with counterfactual of no active case finding intervention. The mass miniature X-ray active case finding campaign occurred between dashed lines (11th March–12th April 1957). CNR: case notification rate. ACF: active case finding. (TIFF) [file pmed.1004448.s012.tiff]

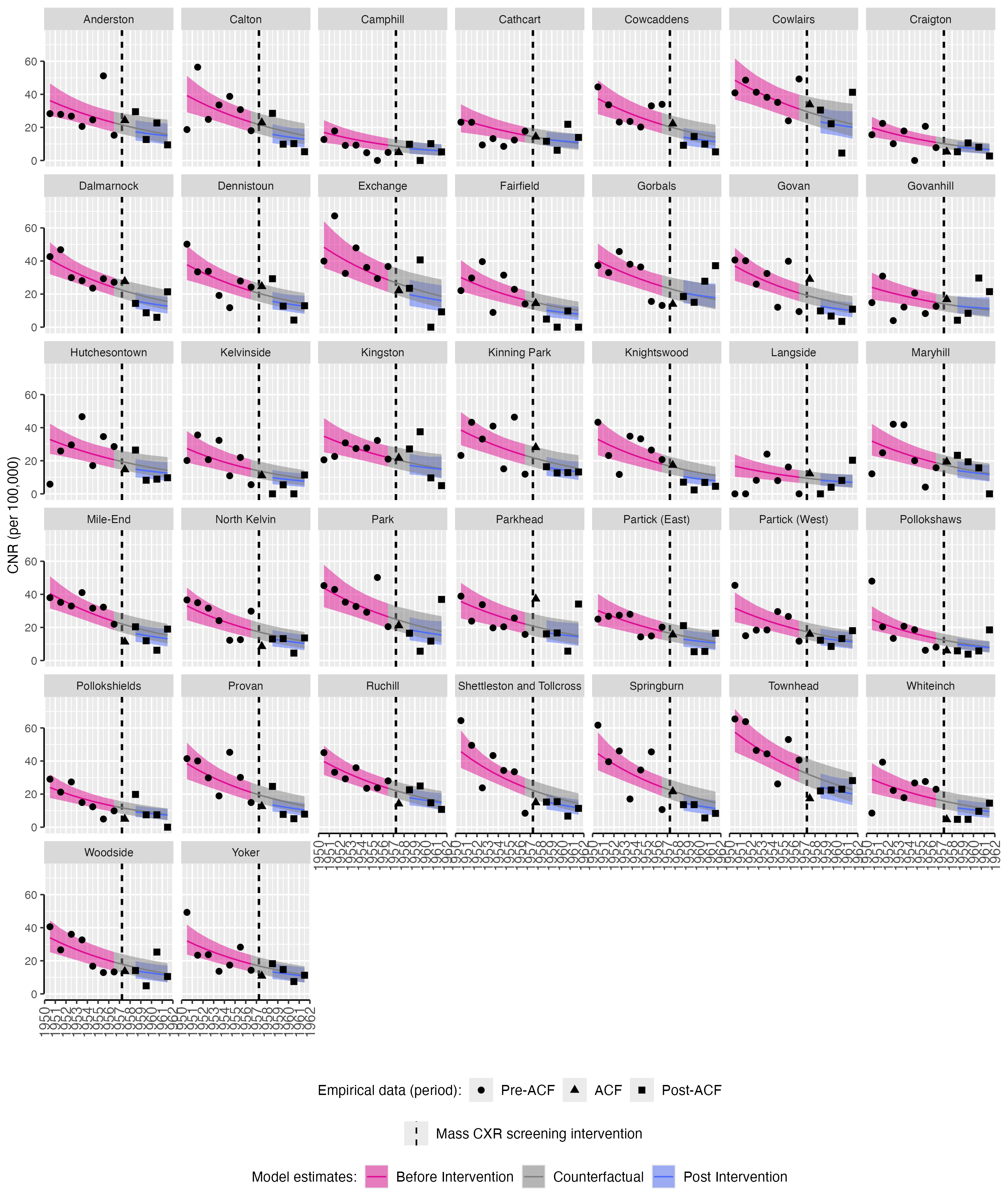

Supplement: S10 Fig — Empirical and modelled case notification rates (per 100,000 population) by ward, with counterfactual of no active case finding intervention. The mass miniature X-ray active case finding campaign occurred between dashed lines (11th March–12th April 1957). CNR: case notification rate. ACF: active case finding. (TIFF) [file pmed.1004448.s013.tiff]

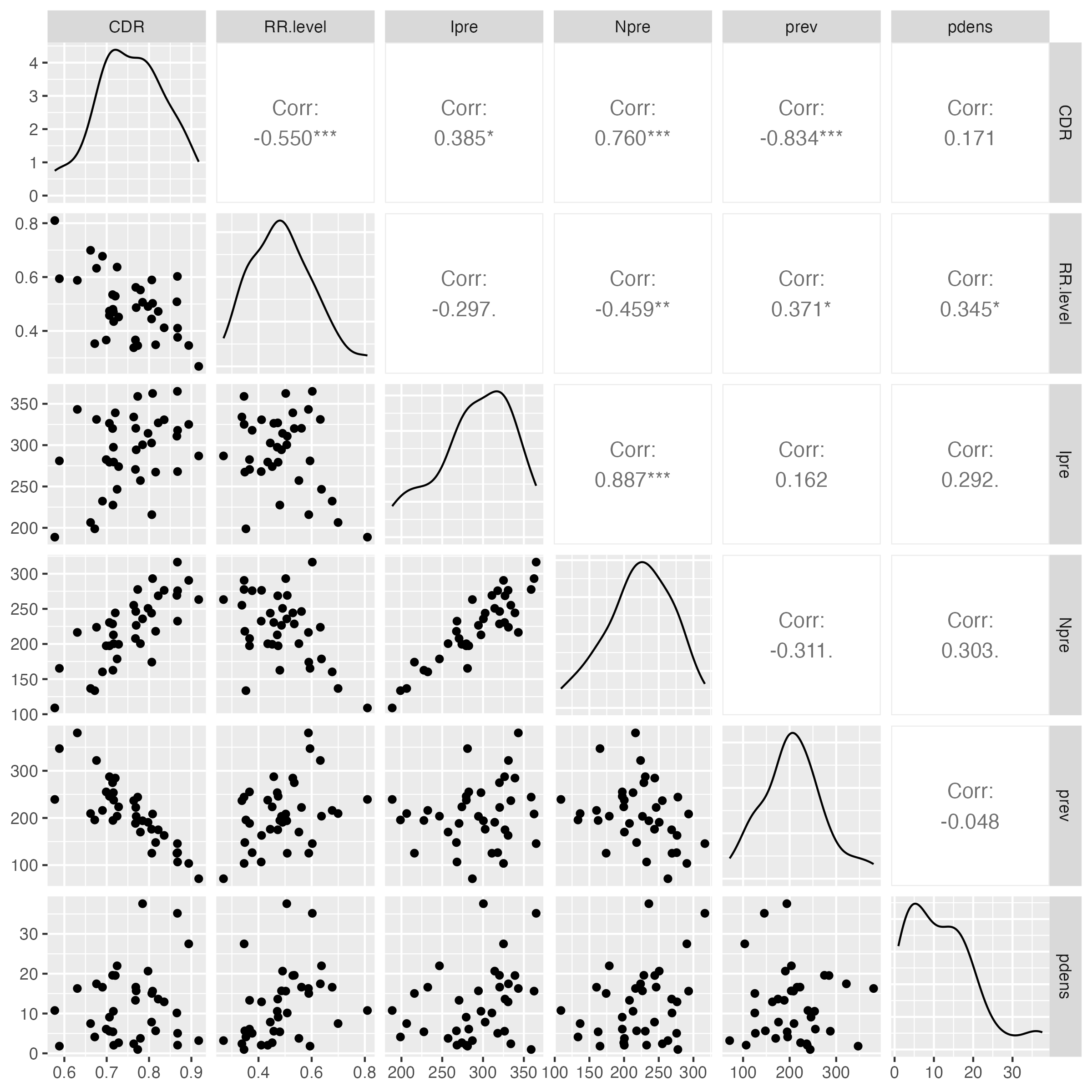

Supplement: S11 Fig — Points are Ward specific values. CDR: Case detection rate; RR.level: Mean posterior relative pulmonary tuberculosis case notification rate in 1958 vs. counterfactual (“level effect”); Ipre: mean estimated incidence per 100,000 in pre-ACF period; Npre: mean case notification rate per 100,000 in pre-ACF period; prev: estimated prevalence per 100,000 in pre-ACF period (1950–1956); pdens: population density (1,000 people per square kilometre). (TIFF) [file pmed.1004448.s014.tiff]

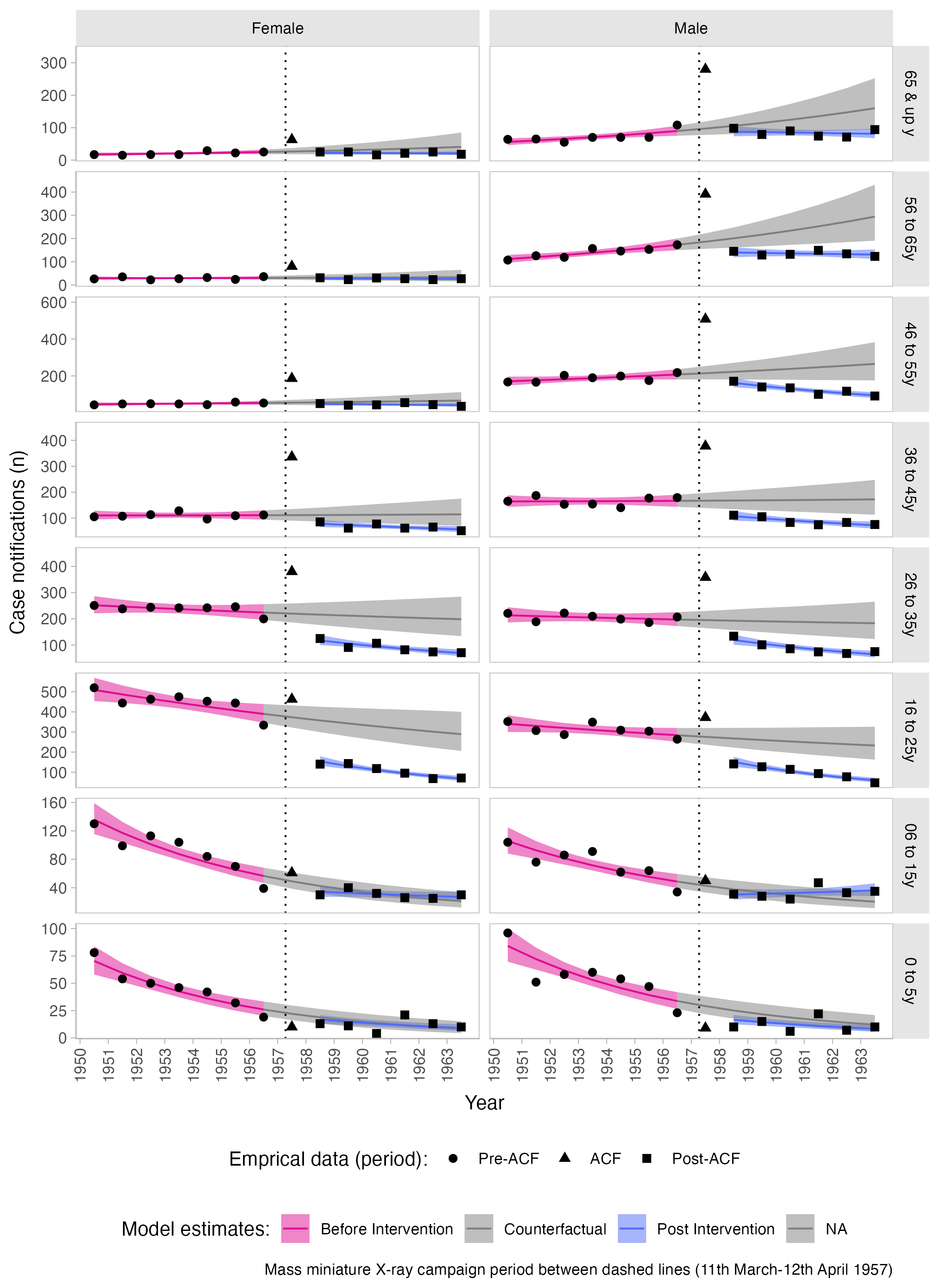

Supplement: S12 Fig — Empirical and modelled case notification rates (per 100,000 population) by ward, with counterfactual of no active case finding intervention. The mass miniature X-ray active case finding campaign occurred between dashed lines (11th March–12th April 1957). CNR: case notification rate. ACF: active case finding. (TIFF) [file pmed.1004448.s015.tiff]
