# Supplementary material for: Impact of active case finding for tuberculosis with mass chest X-ray screening in Glasgow, Scotland, 1950–1963: An epidemiological analysis of historical data
Source: PLoS Med. 2024 Nov 5;21(11):e1004448. doi: 10.1371/journal.pmed.1004448 (PMC11537369; doi:10.1371/journal.pmed.1004448)
Supplement: Attachment — Submitted filename: macpherson.pdf [file pmed.1004448.s016.pdf]

This paper discusses a natural experiment of intensive screening for TB in Glasgow in the last half of the last century.

I was asked for a methodological report and I interpret that to include all aspects of the design and conduct of the study.

Points of detail

Page 5 I was amused to see the use of cigarettes as an incentive in a health study. *O tempora, O mores.*

Page 7 I think we may need a bit more detail about the model either here on in the supplementary equation. Referring to the supplement the last four lines are fine as they just specify priors which get swamped by the data anyway. The four lines prior to that specify a covariance matrix which apart from my not knowing what the parameter 4 means is also fine. However the line

$$V = [Z \quad U] \in \mathbb{R}^{K \times 6} \tag{1}$$

defeats me. I assume $[Z \quad U]$ defines a matrix formed by concatenating Z and U but is it really saying we are dealing with qa matrix with elements in a $6K$–dimensional hyperspace? Perhaps it means this is a K by 6 matrix but in that case why is it specified as $\mathbb{R}$ and where does the 6 come from? I think a sentence could help here explaining in words what role the covariance matrix plays in the formulation in the second line.

Page 8 I can see what the peak effect was but it did not seem to me a particularly intuitive term. To be picky the intervention could not have had its effect over the whole of 1957.

Page 8 I do not think the layout of the equation works well. When I first read it I assumed the −1 was a superscript in the wrong font. On reflection and after comparing with page 40 I think the denominator should be $(\frac{N_{during}}{N_{pre}}) - 1$. If that is correct why not write it as such?

Page 12 Is there any way of putting these into the context of the WHO recommendations on page 3?

Page 13 Looking at Figure 1 the results speak for themselves. I appreciate there is a lot of high level analysis in the background but the plots make the effect very clear.

Page 15 The snag with using caterpillar plots is that the areas do not, in general, come out in the same order making it hard to compare them. If that is the authors' intention then a different form of plot would be needed.

Page 17 Is it possible that readers could complain that all that has happened is that cases have been brought forward by screening and would eventually have happened anyway? This is not quite like lead time bias in cancer screening but is not completely unlike it either. I am not a TB expert and it may be obvious to those who are whether this is an issue.

Page 20 The consistency across wards is impressive and does reinforce the message.

Page 21 and 22 While not affecting the value of the authors' analysis I must confess to a certain scepticism about whether this degree of response would be achieved today in Glasgow or anywhere else globally including TB–endemic areas. To be fair the word speculate does suggest the authors know their results are rooted in a space–time context which may affect generalisability.

Page 23 I might have used the phrase negative control to describe the effect or lack of it on extra–pulmonary.

S5 This could be a bit clearer. Are these sex–specific percentages or overall? The results for young women seem rather anomalous.

S12 This had me confused as I was reading it as a graph where it is conventional for the y–axis to be plotted ascending upwards. The authors obviously view it as a table where the opposite convention is followed. Is that wise?

Summary

Mostly for clarification. I do not think my concerns about relevance to 21st century TB–endemic countries weigh heavily against the paper.

Michael Dewey
