# Supplementary material for: Impact of active case finding for tuberculosis with mass chest X-ray screening in Glasgow, Scotland, 1950–1963: An epidemiological analysis of historical data
Source: PLoS Med. 2024 Nov 5;21(11):e1004448. doi: 10.1371/journal.pmed.1004448 (PMC11537369; doi:10.1371/journal.pmed.1004448)
Supplement: Attachment — Submitted filename: 2024-08-30_glasgow-acf_r1_response.pdf [file pmed.1004448.s017.pdf]

Comments from the Academic Editor

> Thanks for this interesting article. We would need the authors to temper the language a bit though, although epidemiologically the incidence rates are similar in some LMIC now to where Scotland was in the 50s, the diagnostics used in ACF and (treatment available) is very different. So the generalisability will be fairly limited

Author's Response: Thank you for this recommendation. We have reworded our conclusions to temper our language, and emphasise these issues of generalisability and historical applicability to modern times, including emphasising the role of new technologies for screening (e.g. digital chest X-ray).

Lines 69-72:
"Limitations include the lack of data in historical reports on microbiological testing for tuberculosis, and uncertainty in contributory effects of other contemporaneous interventions including slum clearances, introduction of BCG vaccination programmes, and the ending of post-war food rationing."

Lines 92-97:
"A single, rapid round of mass screening with chest X-ray (probably the largest ever conducted) likely resulted in a major and sustained reduction in tuberculosis case notifications. Synthesis of evidence from other historical tuberculosis screening programmes is needed to confirm findings from Glasgow, and to provide insights into ongoing efforts to successfully implement active case finding interventions in today's high tuberculosis burden countries and with new screening tools and technologies."

Comments from the Editor

> On the practices of the times, we wondered how the BCG vaccination may have played a role. If online sources are to be believed, the vaccination program started in 1953 and we wondered if (and how) this may have an impact.

Author's Response: Thank you. We reviewed Medical Officer of Heath reports for Glasgow, which describe the introduction of BCG vaccination programmes. According to reports, BCG was first introduced in the city in 1950 and offered to people with a positive tuberculin skin test and in one of the following four categories: "nurses in hospitals, especially institutions for tuberculosis"; "newborn infants of tuberculous mothers"; "contacts of cases of open tuberculosis"; and medical students. In September-November 1953, this BCG vaccination programme was extended to include school children aged 13 years of age with a positive tuberculin skin test. In 1953, there were 6,648 children aged 13 years of age attending 109 schools in Glasgow who received BCG vaccination. During the mass screening campaign in 1957, tuberculin skin test positive contacts of participants diagnosed with tuberculosis were additionally offered BCG vaccination.

Whilst we agree that it is plausible that the introduction of the BCG vaccination programme may have had a contributory role in slowly reducing tuberculosis notification rates, we believe that BCG vaccination alone is unlikely to be the sole alternative explanation for the epidemiological trends in case notifications observed in the data. Our reasoning is firstly that the BCG vaccination programme in the city had been running for ~7 years prior to the mass screening campaign without an appreciable downwards effect on case notification rates (e.g. between 1950 and 1956: see Figure 1); should the vaccination programme have had a large effect, we would have expected to have seen a signal of this in case notification rates prior to the implementation of the mass screening programme. Secondly, whilst there is some evidence of a small protective effect against infection and development of tuberculosis disease (and hence development of infectious pulmonary tuberculosis with the capacity to transmit to others) for people vaccinated in adolescence and adulthood (particularly at northern latitudes), the greatest benefit of BCG vaccination appears to be to infants at risk of severe tuberculosis disease. Overall, our reading of the literature is that BCG vaccination programmes are unlikely to have been a major contributor to tuberculosis care and prevention efforts, and are very unlikely to have resulted in the substantial

increase in the rate of decline of tuberculosis case notification rates immediately following the mass screening programme.

We have added text to the manuscript to discuss these issues, and emphasise that our understanding of the impact of the BCG vaccination programme on tuberculosis epidemiology remains uncertain.

Lines 861-877:

"According to historical Medical Officer of Health reports, Bacillus Calmette–Guérin (BCG) vaccination was first introduced in Glasgow in 1950 and offered to people with a positive tuberculin skin test and in one of the following four categories: "nurses in hospitals, especially institutions for tuberculosis"; "newborn infants of tuberculous mothers"; "contacts of cases of open tuberculosis"; and medical students. In September-November 1953, this BCG vaccination programme was extended to include school children aged 13 years with a positive tuberculin skin test. During the mass screening campaign in 1957, tuberculin skin test positive contacts of participants diagnosed with tuberculosis were additionally offered BCG vaccination. Although systematic reviews suggest that there is evidence to support a protective effect of BCG vaccination on infection and disease, particularly for younger children [27,28], the greatest protective benefit appears to be to infants at risk of severe tuberculosis disease, who themselves are unlikely to transmit to others. Overall, we believe that the Glasgow BCG vaccination programme was unlikely to have been a major contributor to tuberculosis control efforts, and is unlikely to explain the substantial increase in the rate of decline of tuberculosis case notification rates immediately following the mass screening programme. However, we acknowledge that the combined effects of screening, social improvements, and improved tuberculosis diagnosis and treatment remain difficult to untangle."

Reviewer 1

Comment #1.1: Great to include details on the incentives used in the implementation. Line 405: you do speculate around the role of the high coverage of the screening. It may be worthwhile revisiting this in the discussion with some consideration of what it may take to achieve similar in our current context. The use of incentives in trials has substantial oversight from ethics committees and it is near impossible for me to conceive that an existing Ministry of Health in a high burden setting would be able to budget and implement such an incentive programme.

Author's Response: Thank you. Yes, we agree that this is an important issue. We would frame the extensive programme of engagement and community mobilisation implemented in the Glasgow mass screening campaign as being broader than just "incentives". Indeed, as we describe in the manuscript, we believe that the achievement of high coverage of screening in Glasgow was critically dependent upon the programme of ~12,000 volunteers mobilised to support the delivery of the campaign (e.g. through door-to-door visits), engagement of local businesses, the media and celebrities, and decentralisation of screening units to the local ward-level (with central logistical and operational support). Although we don't have costing data, our impression is that the incentive "prizes" probably only formed a small component of the overall budget of the campaign, but generated a huge amount of positive publicity and support.

Community-based active case finding for tuberculosis is recommended by WHO, and routinely undertaken in many countries around the world, often led by national tuberculosis programmes. Unless a new intervention was being planned, this routine offer of screening would not require approval for research ethics committees, and so we don't feel that ethical review committee oversights of incentives is a major practical barrier to implementation here.

We agree that currently budgets of Ministries of Health are pressed, but community active case finding interventions are likely to be cost-effective, and may be cost saving in the medium term, if implemented efficiently. However, as in the manuscript, we argue that for tuberculosis screening programmes to be effective, they need high levels of community engagement, mobilisation and support. The model of community mobilisation deployed in Glasgow could be implemented in many settings around the world, and may be more effective than “top-down” programmes that are often implemented nowadays. Indeed, this point is emphasised by a recent Cochrane review of qualitative evaluations of tuberculosis screening programmes (referenced in the manuscript), which found local ownership and leadership to be critical determinants of success.

Comment #1.2: This nicely points to potential value of CXR screening beyond TB. It is understandable that more detailed analysis of this is outside of the scope of your article, but a comment in the discussion on this would be very useful. As high burden countries in resource limited settings need to consider costs and benefits, the utility of this programme beyond TB could further support implementation. Perhaps reference to any other work that has projected the additional benefits and opportunities to integrate with more holistic health screening or the need for future analyses that explore this would be good.

Author’s Response: Thank you. We agree that this is an important point. The burden of non-tuberculosis lung disease identified can be expected to be high, and especially so in countries going through demographic transitions and with emerging epidemics of non-communicable diseases. Indeed, in a previous analysis of chest X-rays taken in the 2016 Kenya National tuberculosis Prevalence Survey (*Mungai et al Thorax 2021*), we found a substantial burden of non-tuberculosis lung disease, predominately related to cardiovascular pathology and chronic obstructive airways disease. Similarly, Wong et al found a high prevalence of infectious and non-communicable diseases among adults participating in a tuberculosis prevalence survey in KwaZulu-Natal in South Africa (*Wong et al Lancet Global Health, 2021)*. To the best of our knowledge, there has been very little research beyond this into describing the health needs of people participating

in tuberculosis screening programmes, despite repeated calls from ourselves and other research groups. Recently, we discussed this issue and set out a research agenda in a position piece around new approaches to active case finding for tuberculosis (*MacPherson et al BMC Global and Public Health, 2024*). We have added text to the manuscript to highlight this important point.

Lines 907-915:

"Although mass tuberculosis screening campaigns may be an opportunity to integrate combined health and public health surveillance interventions (as we have previously argued[17]), programmes need to plan for the substantial additional healthcare resources that will likely be required due to detection of other health issues; in the Glasgow campaign, a substantial burden of non-tuberculosis pulmonary disease was identified requiring assessment at city hospital chest clinics. More recent data from Kenya[31] and South Africa[32] emphasises that in countries undergoing health and demographic transitions, the health needs and prevalence of non-communicable diseases in people participating in tuberculosis screening programmes continues to be high."

Comment #1.3: During the ACF intervention - Glasgow almost doubled its case notification in that 1 year. While you have stated you were unable to track and report outcomes, it is essential to describe and consider the ability of a City to effectively deal with a doubling in case notification. In the conclusion you describe the support needed for the implementation of the CXR screening but weak/fragile health systems could be especially vulnerable to a surge in TB diagnoses. Could you consider describing what Glasgow had in place to manage this increase or what a high-burden, resource limited setting may want to put in place to ensure optimum treatment outcomes.

Author's Response: Thank you. Another very important point, and related to Point 1.2 above. We agree that adequate preparation of the health system – and particularly tuberculosis services – is a likely to be a critical determinant of success of mass screening programmes. In response to the Reviewer's comment, we reviewed reports on the implementation of the Glasgow mass screening campaign (Medical Officer of

Health's Report from 1957, and the report on the programme prepared by Sir Kenneth Cowan, and published in 1957).

Whilst it is clear that extensive administrative and committee planning was undertaken for management of people who had a screen-positive miniature chest X-ray, there is surprisingly little details of how this was managed in practice and the impact on day-to-day workload during and after the campaign. Indeed, the city Radiography Section (in charge of administering recall full chest films) reported only that *"… the pressure on work was always considerable, and at times very heavy…"*. The only comment on the impact on hospital referral services that we identified was "*Later the load fell on the chest clinics, and it was necessary to make appointments for Saturdays and Sundays in addition to the increased number of sessions during the week*". To the best of our knowledge, there are no data available (e.g. case fatality rates, treatment completion rates) to indicate whether this increased workload impacted quality of tuberculosis care.

We have added text to the manuscript to emphasise the importance of planning and adequately resourcing health services and tuberculosis to meet increased demand.

Lines 904-907:
"There may additionally be important implications for health systems when planning mass screening campaigns. In the Glasgow campaign, additional chest clinics were required to run during weekdays and at weekends to meet demands of new referrals from the screening programme."

Comment #1.4: Line 55-56: Consider changing to month by name as per main text to eliminate confusion with ddmmyyyy and mmddyyyy.

Author's Response: Thanks. We have done this.

Comment #1.5: Line 389: American should be America

Author's Response: We have corrected this.

Comment #1.6: Line 389: missing is .....evidence, there is a danger........

Author's Response: We have corrected this.

Comment #1.7: Line 471: delete of

Author's Response: We have corrected this.

Reviewer 2

Comment #2.1: The issue of poor bacteriological confirmation during the screening and the lack of a sub-analysis around microbiologically confirmed cases. This depends on the quality of bacteriology available during the day, and it's utilization, which is not quite addressed. [...] What does that microbiological examination consist of? Is there data? [...] With only 22% of detected cases that were bacteriologically confirmed, one wonders, what exactly was being detected and treated? How important, or not important these individuals were to the subsequent impact observed remains untold, and indeed unexplored. Because there were still 1,556 persons routinely diagnosed during the year, presumably because they were sick. One could argue that it doesn't matter, because the screening program happened, they detected and treated these cases, and that was the impact. But because the intervention was mass radiographic screening, involving 37 units and 12,000 volunteers and a whole of society campaign for a paltry 1 million population (one unit and ~300 volunteers for every ~25K population), it does matter. Was the additional impact that is shown very convincingly being driven by the 523 who were (presumably) smear or culture positive, vs the (2369-523=)1846 who

> were apparently bacteriologically negative? Was the reduction in level and slope of notification a direct effect of removing prevalent TB cases and their subsequent transmission, or was it an effect of 'treatment as prevention' to radiographic abnormals, effectively pre-treated a subset of future cases in the campaign year? Was the effect due to reduced transmission, or due to mass prophylactic treatment to a thousands of high risk individuals? Accordingly I'd recommend (a) the notification table 1 (routine and screening) be updated and split by bacteriologic confirmation (or an additional main table added with the stratification - not the supplement, please). (b) a sub-analysis accounting for the pre and post screening impact on bacteriologically confirmed TB be conducted. I don't know if it will have the power to detect. This recommendation is contingent on the bacteriological practices of the day. If the bacteriological confirmation was done by smear only, then this is probably not worth it to do the subanalysis due to insensitivity of detection, and would just add the caveat around bacteriologic confirmation as a limitation in the discussion. [...] There's some reference in the discussion that this might not have been possible. [...] However, I'd suggest that this be included in the methods up front and at least better discussion of what it was that actually might have been driving change.

Author's Response: We very much agree with the Reviewer that greater details of the microbiological testing done for participants in the mass screening campaign (as well as for those notified with tuberculosis through the routine health system between 1950 and 1963) would be extremely useful. However, in the historical records available, there are no detailed reports of microbiological methods or testing results. Indeed, the only summary we have is in Sir Kenneth Cowan's 1957 report on the campaign, which states "*…523 (22%) were bacteriologically-confirmed by isolation of M. tuberculosis.*" Unfortunately, as much as we would like to, we are unable to do any further analysis here!

We did mention this in the Discussion section in our submitted manuscript. However, we have expanded upon this point further.

Lines 917-924:

"There was little description available of microbiological testing results or treatment outcomes, and it is possible that there was over-treatment of tuberculosis in people screened, or indeed treatment of people with very early subclinical tuberculosis which would not have usually been detected and treated. It is unclear what the implications of potential over-treatment, or indeed treatment of early tuberculosis, would be for participants and health systems. Future ACF trials should systematically record individual- and health systems- benefits and harms of participating in tuberculosis screening programmes, and greater research is needed into the effects of screening and treatment of early tuberculosis disease."

Lines 69-72:

"Limitations include the lack of data in historical reports on microbiological testing for tuberculosis, and uncertainty in contributory effects of other contemporaneous interventions including slum clearances, introduction of BCG vaccination programmes, and the ending of post-war food rationing."

Comment #2.2: Update of screening. [...] I think you're trying to say that the effect of ACT3 was actually similar of that observed here, and those differences are interesting and should be explored. On one hand you have a lower sensitivity screening tool (MMR, which is lower than current dCXR can detect), on the other you have molecular testing irrespective of CXR results, including detection of some individuals who may have not had detectable CXR abnormalities. So it's apples and oranges, and I think maybe a bit more nuance about the comparison is warranted.

Author's Response: Thanks, and we agree with the Reviewer's comments here. We have modified this paragraph to add greater nuance here, and to reflect upon the differences between screening approaches, and the potential impact of earlier detection of subclinical tuberculosis through screening.

Lines: 815-837:

"Two contemporary randomised trials of community-based active case finding (ACT3 [15] and TREATS [16]) have shown mixed results, with the more intensive ACT3 study in Vietnam resulting in a substantial reduction in prevalent pulmonary tuberculosis following universal sputum testing with Xpert, whereas no effect was identified from a symptom screening approach in TREATS. We speculate that the very high coverage of chest X-ray screening achieved in Glasgow in 1957 was a major contributor to epidemiological impact, and potentially identified people with early and subclinical tuberculosis, rapidly reducing transmission. This would align with the experiences of the ACT3 trial, where universal sputum testing, as in the Glasgow mass screening campaign, likely identified people in the subclinical state [25]. Greater research to understand the epidemiological impact of the effect of detection and treatment of early states of tuberculosis is needed."

Comment #2.3: Discussion - context needed about secular trends, with more information and a bit more humility in attribution of the changes occurring to the screening campaign. There's no mention of a rather momentous event of the time, which is the post-war lifting of food rations in 1954 in England, or the effect of improved nutritional conditions. That may have had some interaction with the accelerated decline in rates. Which speaks to the question, this was a singled-ended one time event Glasgow. What about places which didn't have such a massive screening campaign, and had to exist with the secular changes of the time? A more thorough evaluation would at least acknowledge this limitation. However, why not actually go farther and propose a more detailed evaluation of the historical evidence of the day, then, comparing other areas? There's a grad student in there, looking for a PhD topic...

Author's Response: Thanks. Again, a very helpful comment, and we are grateful for the insights. Indeed, the end of food rationing in 1954 is likely to be an important event for improving the nutrition of the people most at risk of tuberculosis, and occurred only a few years earlier than the mass screening campaign. Given what we now know about the effect of improving nutrition on risk of tuberculosis (e.g. the RATIONS trial), this is

likely to have some contributory impact on declining notification rates. As we noted in the manuscript Discussion section, there were likely multiple factors that contributed the long-run decline in tuberculosis notifications (and potentially could have resulted in an accelerated decline contemporaneous with the mass screening campaign). However, given the rapidity and magnitude of effect, and consistency between all 37 city wards, we think it plausible that the campaign did have a substantial impact, and probably was the most important factor in changing the epidemic trajectory. Nevertheless, we have reworded our conclusions to emphasise greater uncertainty here. We have additionally added text to the discussion section to highlight the importance of nutrition.

We very much agree that future comparative research with a larger set of historical datasets (and particularly from a variety of international settings) would be insightful (and indeed a very enjoyable PhD project!). We have added text to the Discussion to emphasise this point.

Lines 958-960:
"Synthesis and comparative analysis of other historical mass tuberculosis screening programmes could give insights into the magnitude of effectiveness of community-based active case finding programmes."

Lines 69-72:
"Limitations include the lack of data in historical reports on microbiological testing for tuberculosis, and uncertainty in contributory effects of other contemporaneous interventions including slum clearances, introduction of BCG vaccination programmes, and the ending of post-war food rationing."

Comment #2.4: Age and sex effects. [...] This is a bit hard to swallow from the data presented, which appear to have been drawn from very small numbers, for the young children argument. the numbers averted suggested that the rate change was applied to a very small number of cases both before and after the intervention. Perhaps please

> provide the numbers of notification, and the rate among kids, in the pre and post period. Birth rates may have also been expanding in the postwar era, inflating the infant denominator. Perhaps this also needs to be couched with the unmentioned counterpoint, that there was an increase in slope of notifications in the 6-15 age group. So it's not really clear what's 'strong' evidence here. Possible evidence, maybe.

Author's Response: Thanks. This is a good point, and we apologies for overstating here. Numbers of notifications for each age group (by year and sex) can be seen in Figure S13 (was Figure S12 in previous version; note that the y-axis is absolute numbers of notified cases, rather than case notification rates). The population pyramids shown in Figure S3 show little change in the numbers of children aged 0 to 4 years of age between 1950 and 1963, indicating that the infant denominator is unlikely to be of major importance. We have reworded this text to appropriately reflect our uncertainty here.

Lines 879-891:
"We found evidence that the impact of the ACF intervention differed by age group and sex. In young children (<5 years, who themselves weren't eligible for screening, although a small number of children <15 years did undergo chest X-ray), case notification rates decreased during the intervention year in contrast to other age groups which saw large increases; we speculate that this may indicate evidence of ACF shortening infectious duration and providing early beneficial impact on transmission."

> **Comment #2.5:** Conclusions [...] This conclusion may need to be couched in less certain causality, given the uncertainties in secular trends. It's likely to have resulted.

Author's Response: Thank you. We agree, and have revised the conclusions to reflect this uncertainty.

Lines 69:72:

"Limitations include the lack of data in historical reports on microbiological testing for tuberculosis, and uncertainty in contributory effects of other contemporaneous interventions including slum clearances, introduction of BCG vaccination programmes, and the ending of post-war food rationing."

Lines 92-97:

"A single, rapid round of mass screening with chest X-ray (probably the largest ever conducted) resulted in a major and sustained reduction in tuberculosis case notifications. Synthesis of evidence from other historical tuberculosis screening programmes is needed to confirm findings from Glasgow, and to provide insights into ongoing efforts to successfully implement active case finding interventions in today's high tuberculosis burden countries and with new screening tools and technologies."

Reviewer 3 (Statistical reviewer)

Comment #3.1: I was amused to see the use of cigarettes as an incentive in a health study. O tempora, O mores.

Author's Response: We were likewise interested to find this in the historical reports, and certainly wouldn't recommend as an incentive for a public health campaign now!

Comment #3.2: I think we may need a bit more detail about the model either here on in the supplementary equation. Referring to the supplement the last four lines are fine as they just specify priors which get swamped by the data anyway. [...]I think a sentence could help here explaining in words what role the covariance matrix plays in the formulation in the second line.

Author's Response: Thanks for this suggestion, we had perhaps over-relied on the equations and have now added some additional explanatory text beneath Equation 1 in

the Supplemental Material (S1 Text), which we hope helps with your specific request for detail as well as aiding interpretability.

"The first line represents the data likelihood, with the data modelled as following a negative binomial distribution, with a single dispersion parameter ϕ modelled with a gamma prior. The second line models the mean of the negative binomial distribution in terms of global effects representing the level and trends during the 3 ACF periods (α and β respectively), as well as ward-level random effects in the levels (the matrix Z) and trend (the matrix U). The population offset term, $\log(N_i)$, ensures we are modelling per capita rates. The intercept (β) and slope (α) coefficients are modelled as having normal priors. The remaining equations jointly model the random effects for the levels and slopes in each ward during each of the 3 ACF period as a 6-dimensional multivariate normal (MVN). This approach allows for correlations between the level and slope random effects across ACF periods. The mean for top level MVN is modelled as having an iid normal prior, and the MVN covariance matrix is modelled using the Lewandowski-Korowicka-Joe (LKJ) distribution recommended for modelling covariances in Stan (the distributions specified for Σ, and the additional random variables Ω and τ_k)."

Comment #3.3: I can see what the peak effect was but it did not seem to me a particularly intuitive term. To be picky the intervention could not have had its effect over the whole of 1957

Author's Response: Thanks. In using the term "peak effect", we intended to convey the peak in annual case notification rates, as this outcome is measured at yearly resolution. Whilst we acknowledge that the "peak" probably occurred in the months following the intervention, there are no data available to confirm this. We tried using other terms (e.g. intervention effect, ACF effect), but felt that all suffered from the same issue. Therefore, for the purpose of comparing between years, we think it reasonable to call the model-predicted 1957 estimates the "peak" effect, facilitating comparison between study years.

Comment #3.4: I do not think the layout of the equation works well. When I first read it I assumed the −1 was a superscript in the wrong font. On reflection and after comparing with page 40 I think the denominator should be (Nduring) − 1. If that is correct why not write it as such?

Author's Response: Thank you for carefully checking the equations and drawing attention to the potential for misreading as previously formatted in the main text. The denominator is correct as written, and corresponds with the equation in the Supplemental Material (and associated working above). However, we have re-written the equation in the main article to avoid confusion associated with type-setting.

Lines 280-282:

$$odds(CDR) = \frac{T \times cov}{(R - 1)}$$

Here, $R = N_{during}/N_{pre}$ is the ratio of notification rates during vs before the intervention, T is the ...

Comment #3.5: Page 12 Is there any way of putting these into the context of the WHO recommendations on page 3?

Author's Response: On Page 12, we report ward-specific pulmonary case notification rates for all ages, whereas WHO recommendations are based on adult (>15 years) pulmonary TB prevalence thresholds. However, through our equilibrium competing hazards model, we do estimate ward-specific prevalence rates in the pre-ACF period; these results can be seen in Figure S13 (panels indicated with label ` prev` : we have expanded the footnote description to make clearer).

Of note, from this model, all wards have an estimated pre-ACF prevalence of <400 per 100,000 people, below the WHO's current conditional recommendation for ACF to be conducted in communities where adult pulmonary tuberculosis prevalence is >500 per 100,000 people. Stimulated by the Reviewer's comment, we think this is a potentially

important point that we didn’t sufficiently highlight in our previous version of the manuscript. We have therefore added text to the Discussion to emphasise that, in the period before the commencement of the ACF campaign, estimated prevalence was likely below levels recommended by WHO in all wards, and yet large effects were seen on pulmonary tuberculosis case notification rates. We advance this point to suggest that, if analysis of other historical datasets show similar trends, this might argue for a further lowering of the WHO tuberculosis screening threshold.

Lines 822-835:
“We additionally found that, in the pre-ACF period (1950-1956) all Glasgow wards had an estimated tuberculosis prevalence of <400 per 100,000. This is below the current WHO screening threshold of >500 per 100,000, and suggests that epidemiological impact from ACF may be achieved in settings with more concentrated epidemics; this needs confirmation through analysis of a greater number of historical and contemporary datasets. Further research to understand the epidemiological impact of the effect of detection and treatment of early states of tuberculosis is also needed.”

Comment #3.6: Page 13 Looking at Figure 1 the results speak for themselves. I appreciate there is a lot of high level analysis in the background but the plots make the effect very clear.

Author’s Response: Thank you. We worked hard on these figures to ensure they conveyed the main study messages, and are glad you found them helpful.

Comment #3.7: Page 15 The snag with using caterpillar plots is that the areas do not, in general, come out in the same order making it hard to compare them. If that is the authors’ intention then a different form of plot would be needed.

Author’s Response: Thanks. These plots are ordered by the central effect estimate for each ward, within each outcome (“peak”, “level”, “slope”) group. Our intention was to

compare variation in effects between wards for each measure, rather than within wards. We additionally used the diverging colour scale to indicate the magnitude of ward effects, and well as providing a ward number linking to the maps above to allow identification of spatial patterns in effects. In response to the Reviewer's comment, we tried an alternative figure with the caterpillar plots (panels B1, B2, B3) ordered by ward number. However, we found that this resulted in a considerably more "messy" plot, which would not allow a reader to perceive patterns of variation across the three effects, and which had the consequence that readers would have to rely upon comparing relative position on the x-axis and colour intensity to identify the wards with the greatest (or least) effects. Therefore, if it is acceptable to the reviewer and editor, we would prefer to keep this plot in its current design.

Comment #3.8: Is it possible that readers could complain that all that has happened is that cases have been brought forward by screening and would eventually have happened anyway? This is not quite like lead time bias in cancer screening but is not completely unlike it either. I am not a TB expert and it may be obvious to those who are whether this is an issue.

Author's Response: We agree that detection of tuberculosis cases will have likely been brought forward in time. As tuberculosis is an infectious disease transmitted between people, this has important implications that are not seen with non-communicable disease programmes, such as cancer screening. Shortening the infectious period (by earlier detection through screening), and detection at an earlier, less infectious stage of disease (or indeed before people recognise themselves to be unwell and seek healthcare), will likely result in fewer transmission events, accelerating overall declines in incidence and prevalence. We show this where we estimate a substantial overall reduction in cases in post-ACF period compared to the counterfactual where the programme had not been implemented. We alluded to this in the Discussion where we stated *"We speculate that the very high coverage of chest X-ray screening achieved in Glasgow in 1957 was a major contributor to epidemiological impact, and potentially*

identified people with early and subclinical tuberculosis, rapidly reducing transmission." We have reworded this text to make our point more clearly.

Lines 813-835:
"Two contemporary randomised trials of community-based active case finding (ACT3 [15] and TREATS [16]) have shown mixed results on tuberculosis prevalence and infection, with the more intensive ACT3 study in Vietnam resulting in a substantial reduction in prevalent pulmonary tuberculosis following universal sputum testing with Xpert, whereas no effect was identified from the symptom screening approach in TREATS. We speculate that the very high coverage of chest X-ray screening achieved in Glasgow in 1957 was a major contributor to epidemiological impact, and potentially identified people with early and subclinical tuberculosis, rapidly reducing transmission. This would align with the experiences of the ACT3 trial, where universal sputum testing, as in the Glasgow mass screening campaign, likely identified people in the subclinical state [25]. We additionally found that, in the pre-ACF period (1950-1956) all Glasgow wards had an estimated tuberculosis prevalence of <400 per 100,000. This is below the current WHO screening threshold of >500 per 100,000, and suggests that epidemiological impact from ACF may be achieved in settings with more concentrated epidemics; this needs confirmation through analysis of a greater number of historical and contemporary datasets. Further research to understand the epidemiological impact of the effect of detection and treatment of early states of tuberculosis is also needed."

Comment #3.9: Page 20 The consistency across wards is impressive and does reinforce the message.

Author's Response: Thanks. We agree with this, and think that the ward-level analysis is a key strength of our manuscript.

Comment #3.10: Page 21 and 22 While not affecting the value of the authors' analysis I must confess to a certain scepticism about whether this degree of response would be achieved today in Glasgow or anywhere else globally including TB–endemic areas. To be fair the word speculate does suggest the authors know their results are rooted in a space–time context which may affect generalisability.

Author's Response: We agree with this point, which is similar to points made by Reviewer #2 above. We have edited throughout the text to convey this point.

Comment #3.11: Page 23 I might have used the phrase negative control to describe the effect or lack of it on extra–pulmonary.

Author's Response: This is a reasonable suggestion, and we thank the reviewer for making it. However, phrasing extra-pulmonary tuberculosis case notification rates as a "negative control" may raise concerns within the tuberculosis epidemiology research community. This is because the processes generating pulmonary and extra-pulmonary case notifications are not completely independent (force of infection, population age distribution and characteristics, diagnostic and post-mortem examination capabilities, treatment availability and effectiveness). Thus, our feeling is that the extra-pulmonary tuberculosis case notification rates provide useful comparative data, we would not consider these to be a true negative control. As such we would prefer not to use this term if possible.

Comment #3.12: S5 This could be a bit clearer. Are these sex–specific percentages or overall? The results for young women seem rather anomalous.

Author's Response: These are overall percentages. We agree that the results for young women in the pre-ACF period are high, but this probably reflects known barriers to tuberculosis diagnosis among men, as we discuss in the manuscript.

Comment #3.13: S12 This had me confused as I was reading it as a graph where it is conventional for the y–axis to be plotted ascending upwards. The authors obviously view it as a table where the opposite convention is followed. Is that wise?

Author's Response: Thanks. We have now reordered the age categories so that oldest is at the top of the figure.

Comment #3.14: Mostly for clarification. I do not think my concerns about relevance to 21st century TB–endemic countries weigh heavily against the paper.

Author's Response: Thanks for this comment, and upon reflection, we agree about not extrapolating too much from historical data to current epidemics. As such, we have reworded our conclusion (see also response to comment from the Academic Editor, and Reviewer 2).

Lines 92-97:
"A single, rapid round of mass screening with chest X-ray (probably the largest ever conducted) likely resulted in a major and sustained reduction in tuberculosis case notifications. Synthesis of evidence from other historical tuberculosis screening programmes is needed to confirm findings from Glasgow, and to support efforts to successfully implement active case finding interventions in today's high tuberculosis burden countries and with new screening tools and technologies."
