# Supplementary material for: Impact of active case finding for tuberculosis with mass chest X-ray screening in Glasgow, Scotland, 1950–1963: An epidemiological analysis of historical data
Source: PLoS Med. 2024 Nov 5;21(11):e1004448. doi: 10.1371/journal.pmed.1004448 (PMC11537369; doi:10.1371/journal.pmed.1004448)
Supplement: Attachment — Submitted filename: 2024-09-30_glasgow-acf_r3_response.docx [file pmed.1004448.s018.docx]

**Comments from the Academic Editor**

Author summary: Please consider revising the third bullet point in the ‘What did the authors do and find’ section as follows (adding ‘rates’ before ‘doubled’ and adding a comma after ‘Post-intervention’: “Before the mass screening intervention (1950-1956), tuberculosis notification rates were declining at 2.3% per year, and rates doubled in the year of the intervention (1957). Post-intervention, tuberculosis notification rates declined at 5.4% per year, and there were an estimated 4,599 pulmonary notifications averted.”

**Author’s Response:** Thank you. We have made this change.

Please ensure that the study is reported according to the STROBE (or appropriate STOBE extension) guideline and include the completed STROBE (or STROBE extension) checklist as Supporting Information. Please add the following statement, or similar, to the Methods: "This study is reported as per the Strengthening the Reporting of Observational Studies in Epidemiology (STROBE) guideline (S1 Checklist)." When completing the checklist, please use section and paragraph numbers, rather than page numbers.

**Author’s Response:** Thank you. We have completed the STROBE Checklist, and attached as S1 Checklist.

To help us extend the reach of your research, please provide any X (formerly known as Twitter) handle(s) that would be appropriate to tag, including your own, your co-authors’, your institution, funder, or lab. Please enter in the submission form any handles you wish to be included when we post about this paper.

**Author’s Response:** We have stopped using X (formerly Twitter), as the content being posted and advertised was not conducive to scientific discourse. Instead, we are using Bluesky (@petermacp.bsky.social; @rachaelburke.bsky.social; @hr-stagg.bsky.social). Would be great if PLOS Medicine had an active profile on Bluesky!
